# The Cost Effectiveness of Single-Patient-Use Electrocardiograph Cable and Lead Systems in Monitoring for Coronary Artery Bypass Graft Surgery

**DOI:** 10.3389/fcvm.2019.00061

**Published:** 2019-05-10

**Authors:** Rhodri Saunders, Julie Lankiewicz

**Affiliations:** ^1^Coreva Scientific, Königswinter, Germany; ^2^Cardinal Health, Mansfield, MA, United States

**Keywords:** surgical wound infection, electrocardiography, cross-contamination, postoperative period, patient readmission, patient safety, medical economics

## Abstract

**Background:** During admission for coronary artery bypass graft (CABG) surgery patients receive electrocardiograph (ECG) monitoring; for which reusable ECG cable and leads (rECG) are standard.

**Objective:** Evaluate the cost effectiveness of a single-patient-use ECG cable and lead system (spECG).

**Methods:** Review of the Medicare 2011–2014 database followed by a cost-effectiveness model considering a Medicare facility transitioning from rECG ($9 per patient) to spECG ($15). In-hospital ECG monitoring was for ≤8 days. In the model, patients underwent CABG and recovered in the intensive care unit, before transfer to the general ward and discharge. Surgical site infection (SSI) resulted in increased length of stay, readmission, or outpatient care. Health outcomes impacted EQ-5D-measured quality adjusted life years (QALYs). Health and cost outcomes were discounted at 3.5% annually. All costs in 2016 USD. Significance (95% level) was assessed via 2,000 simulations.

**Results:** In 2014, 5.49% of patients had an SSI by 90-days post-surgery, with spECG reducing the odds of an SSI (odds ratio: 0.74, 0.62–0.89). Mean 40-year, per-patient costs to Medicare were $65,497 with rECG and $65,048 with spECG. The $450 saving was significant, with a median (95% credible interval) reduction of $466 ($174 to $989). Cost drivers were days required to treat inpatient SSIs. QALYs increases with spECG were significant but minor (median increase 0.008). Medicare savings may total $40 million per year with use of spECG.

**Conclusions:** Post-operative SSI is a concern for Medicare patients undergoing CABG, and use of spECG is likely to provide cost and patient benefits.

## Introduction

Hospital-acquired infections (HAI) pose a substantial and growing burden to both patients and providers. Such infections related to cardiac surgery can be extremely burdensome. Mazzeffi et al. found that almost half of adult patients that had an extended length of intensive care unit (ICU) stay following cardiac surgery had one or more HAIs (1). The authors found that developing an HAI more than doubled the probability of patient in-hospital mortality (29 vs. 13%) (1). Surgical site infections (SSIs) further increased the risk of in-hospital mortality to 39.4% (1), a result aligned with multiple other clinical studies from North America (2, 3).

SSIs are a major contributor to the overall burden of HAI and reducing their incidence is a priority for the US Department of Health and Human Services (4). SSIs have been found to complicate between 3.3 and 20.8% of coronary artery bypass grafting (CABG) procedures (3, 5–8). The double-blind clinical trial by Dhadwal and colleagues resulted in 10.4 and 7.6% of the intention-to-treat control group developing superficial and deep SSIs, respectively (7). A 2013 impact analysis found that SSIs resulted in increased mean ICU stay (+1.6 days), non-ICU stay (+3.4 days), total charges (+$22,995), and 30 day readmissions (+43.8 readmissions per 100 procedures) (5). The mean charge per readmission following cardiac surgery was reported to be $39,136 (9). The impact of SSI on healthcare utilization is clear. The patient impact is also substantial, Cohen et al. determined that mean quality of life (where 1.0 is perfect quality of life) at the point of CABG and at 1-year post procedure was 0.74 and 0.85, respectively (10). A systematic review of utilities associated with SSIs found that quality of life was reduced by between 0.04 and 0.48 points per SSI event (11).

In addition to improving patient outcomes, reducing the incidence of SSIs following CABG would likely reduce costs and overall burden on the healthcare system. Electrocardiograph (ECG) monitoring is indicated for all patients undergoing CABG for the duration of hospitalization, but standard of care reusable ECG lead wires (rECG) have been linked to increased risk of infection. Literature indicates that between 51 and 77% of rECG are contaminated with potentially harmful pathogens (12–14). It was hypothesized that single-patient ECG leads and wires (spECG) would reduce the incidence of SSI by limiting the possibility of cross-contamination. Albert et al. compared rECG and spECG, finding no difference in infections (not just SSI) acquired in the ICU, though noting that implementation of additional infection control measures drastically reduced HAI rates during the course of the study (15, 16). More recently, a Medicare claims analysis observing over 27,000 CABG surgeries found that facilities using spECG (Kendall™ DL, Cardinal Health, Dublin, OH) had a 25% reduction (*p* > 0.05) in SSIs at 90 days post procedure (6). In addition to reducing the SSI rate, use of the same spECG has been associated with a lower rate of false alarms and a significantly lower rate of false “leads-off” alarms (16).

In this analysis, a computational model is implemented to explore the potential health economic impact of switching from rECG to spECG within the Medicare patient population.

## Methods

### Clinical Data and Sample Size Determination

Assuming a 5.5% SSI incidence at 90 days, we calculated that a sample size of 17,638 patients would be required to detect a 15% reduction in this rate with alpha and beta error set at 0.9 and 0.8, respectively. In 2018, Lankiewicz et al. published a retrospective, case-controlled, Medicare claims analysis comparing 42 hospitals using spECG (4,450 CABG cases) to 274 hospitals using rECG (22,846 CABG cases) (6). Our analysis builds on this study and its preliminary work using the Medicare Fee for Service Claims database, 2011–2014. This preliminary work included a review of CABG cases in patients aged ≥ 65 years in the Medicare Fee for Service Claims, 2011–2014 five percent professional sample, and an interrupted time series analysis across 5,402 CABG surgeries at 18 acute care hospitals exploring SSI rates through 90-days at facilities that during this time switched from rECG to spECG. The ICD-9 codes used to identify CABG procedures for both analyses were: 36.10 to 36.17 and 36.19, with suspected SSI identified via ICD-9 codes: 998.50, 998.51, and 998.59. Finally, the Mantel-Haenszel odds ratio (OR) and relative risk (RR) was calculated for the full analysis presented by Lankiewicz et al. (6).

As this was a retrospective, deidentified computational simulation analysis, Institutional Review Board (IRB) and standard biosecurity review were not applicable.

### Data Identification

Structured literature review of PubMed was performed on October 18, 2017 to identify data for the cost-effectiveness analysis that could not be extracted from the Medicare database. In searches of PubMed, a total of 24,000 electrocardiogram and 328,228 CABG, coronary surgery and ICU studies were identified before being further refined by relevance, publication recency and key clinical variables included in the abstract, title, and keywords. The methodology for this is shown in Table S1. In total, searches yielded 790 publications of potential interest and likely suited to data extraction for this study. Following full-text screening, key clinical variables of interest were extracted: the cost of resources associated with CABG, intensive care unit stay, hospital stay, and hospital readmission; the incidence and cost of adverse events associated with ECG monitoring; difference in adverse event rates between rECG and spECG; health-related quality of life (EQ-5D or SF-36) associated with patients receiving CABG; quality of life disutilities for adverse events associated with CABG. Further structured literature review detail including an example search for ECG data is provided in Table S1.

### Cost-Effectiveness Analysis

**Figure 1:**
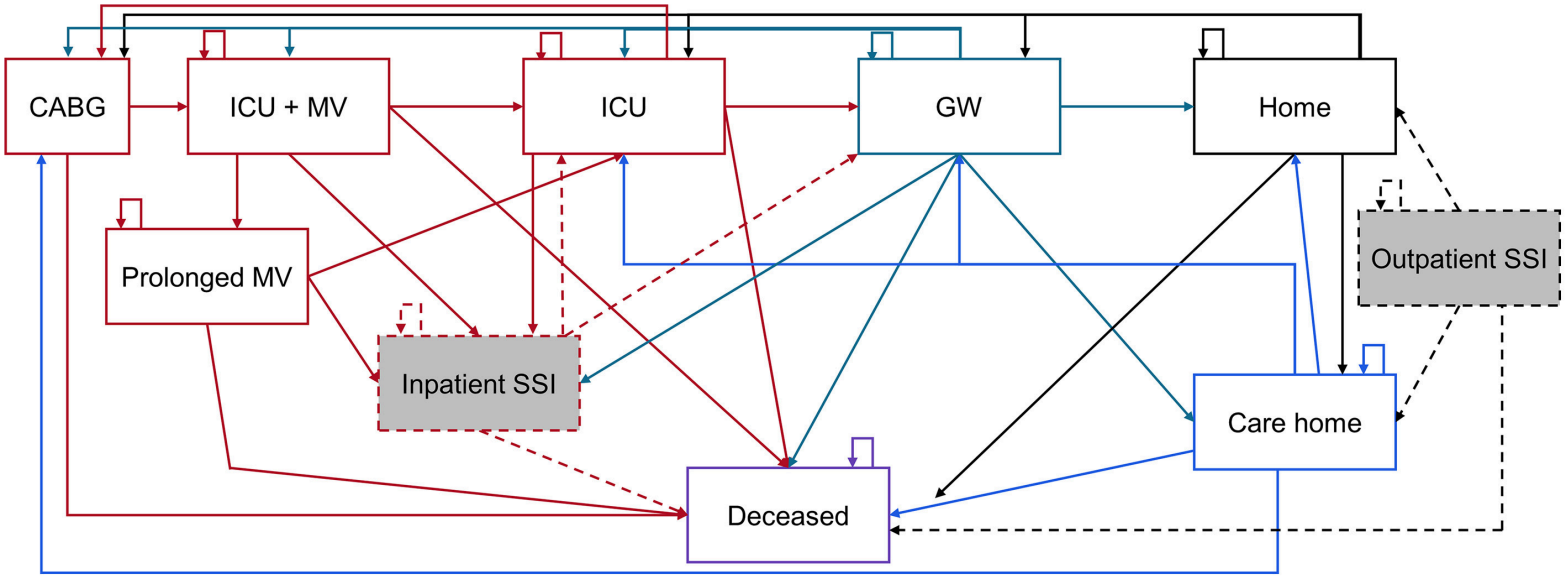
Representation of health states associated with CABG care. CABG, Coronary artery bypass grafting; GW, General ward; ICU, Intensive care unit; MV, Mechanical ventilation; SSI, Surgical site infection. The Markov model is color coded as: Red items are acute care, green = general ward, black = home, blue = care home, and purple = deceased. Shaded gray boxes are SSI states, of which a percentage are deep sternal wound infections.

The cost-effectiveness analysis was developed as a Markov model in Microsoft Excel® with cycle lengths of 1 day to day 91, after which point quarterly (91.3 day) cycles are used up to a time point of 40 years. In a Markov model, patients are in one of a number of discrete health states. The patient cohort will enter the model at health state “CABG,” representing the day of the procedure. “CABG” is the only health state that cannot be directly re-entered, thus multiple CABG procedures within 1 day are not feasible. After 91 days, no repeat CABG procedure is considered. This is supported by the results of the SYNTAX trial, that found only 0.1% of CABG patients had a repeat procedure over 5 years and all occurred in year 1 (10). All other “health states” (Figure 1) can be directly re-entered, i.e., a patient can spend more than one consecutive day in these health states.

From “CABG,” only “deceased” and “ICU + MV” (in the ICU and on mechanical ventilation), are accessible (Figure 1). The standard of care pathway from “ICU + MV” is to remain in the ICU without MV (“ICU”), then be transferred to the general ward (“GW”) and discharged “Home” or to a “Care home.” Alternatively, some patients may remain on MV longer than 2 days and enter the “Prolonged MV” health state, before later rejoining the standard care pathway described above. In the inpatient or outpatient setting, an SSI can develop that requires treatment. Inpatient SSI requires treatment which extends the length of the hospital stay, whereas outpatient SSI can be mostly treated in the outpatient setting. A certain percentage of SSIs are deep sternal wound infections (DSWIs), if in the outpatient setting these are associated with a hospital readmission. All health states can lead to death, but the probability of accessing “deceased” is dependent on the patient's current health state. Each health state is associated with costs and quality of life. These are discounted at 3.5% annually after the first year of the simulation (17). Costs are in 2016 United States Dollars ($), with quality of life measured using utilities from the EuroQoL 5-dimensions questionnaire (EQ-5D) or equivalent tools.

The probability of moving from one health state to another is governed by clinical data taken from peer-reviewed published literature. These rates are adjusted based on certain patient and ECG monitoring factors. These include obesity and diabetes, which is linked to increased likelihood of DSWI and mortality, as well as a longer length of stay (18, 19). The key default model parameters, including the patient population, transition probabilities, and risk factors, are shown in Table 1, the full list is provided in Table S2.

**Table 1:** Model base-case parameters.

**Parameter**	**Base case**	**Variance**	**Reference**
**PATIENT COHORT**			
Age, years	73	2.3	(20)
Gender, % female	29.8	0.18	(20)
Obese, %	35	0.48	(18)
Morbid obesity, %	6	0.24	(18)
Diabetes, %	45.2	0.19	(20)
**CLINICAL SETTINGS**			
Time on MV, days	0.5	0.0001	(19)
ICU time, days	1	0.0001	(19)
Hospital time, days	8	1.79	(20)
Prolonged MV patients	10.6	0.0001	(18)
ECG monitoring, days	8	1.79	(20)
Home discharge	74.5	0.17	(20)
In-hospital mortality, %	2.7 (after 13 days)	0.067	(20)
DSWI, % of SSIs	40.75	2.6	(4)
SSI additional length of stay, days	13.3	20.8	(21)
DSWI additional length of stay, days	24	5	(22)
False alarms, *N* per 100 patient days	97.9	1.43	(16)
Leads-off alarms, *N* per 100 patient days	40.9	4.92	(16)
**RELATIVE RISK OF EVENTS**			
spECG false alarms, RR	0.81	0.13	(16)
spECG leads-off alarms, RR	0.71	0.15	(16)
DSWI RR, BMI morbidly obese	6.45	0.40	(18)
Hospital mortality RR, morbidly obese	1.64	0.18	(18)
Diabetes RR, DSWI	1.71	0.20	(19)
**COSTS**			
rECG cost per patient use	9.08	1.66	†
spECG purchase cost	15	2	‡
CABG	10,244	2,664	(10)
Mechanical ventilation	756	210–906	(23)
ICU per day	2,536	2,197–3,066	(23)
Nurse time, $ per hour	58	4.6	(24)
Inpatient SSI^*^	−158	210	(5)
Outpatient care for SSI	2,583	838	(25)
Readmission for DSWI	23,586	6,815	(9)
Future care costs	4673.1	524	(10)
Decrement, future care costs	92.29	215	(10)
**QUALITY OF LIFE**			
Baseline	0.85	0.158	(10)
CABG	0.741	0.191	(10)
Mechanical ventilation	−0.39	−0.59–0.09	(26)
ICU stay	0.402	0.36–0.44	(27)
General ward stay	0.52	0.45–0.59	(28)
SSI	0.198	0.04–0.8	(11, 29)

*Costs are in 2016 USD, with conversion from the published figure using the CPI for medical care (Series Id: CUUR0000SAM)*.

* *Surgical site infection is generally found to increase hospital length of stay and hospital costs, but reduce the mean cost per day in hospital*.

*^†^Calculated value ‡Data on file with Cardinal Health*.

The base case analysis used the default values as input. The significance of this result was assessed using 2,000 probabilistic sensitivity analyses. In each analysis, every model parameter was individually sampled and the model result recalculated. For sampling, a random number between 0 and 1 was generated and used as the input of the cumulative probability function. The cumulative probability function had a lower limit (0) of the lower range of the variance (see Table 1) and an upper limit (1) of the upper range of the variance. Most parameters were sampled from a normal distribution, except for relative risks that used a log-normal distribution. From the 2,000 simulations the 95% credible interval (CrI) was estimated, the range in which 95% of results falls. The CrI was used instead of the confidence interval (CI) because the model results did not follow a normal distribution.

## Results

### Clinical Data

Medicare Fee for Service Claims, 2011–2014 five percent professional claims sample included a total of 17,182 CABG patients. The percentage of patients experiencing an SSI by 30-days post procedure showed a slight decrease over the time frame: 3.66% (2011), 3.65% (2012), 3.43% (2013), and 3.44% (2014). A similar trend was seen in the 90-day post procedure SSI rate: 6.49% (2011), 5.78% (2012), 6.00% (2013), and 5.49% (2014). During the period 2011 to 2014, 18 reporting Medicare facilities (100% sample) switched from rECG to spECG. Overall these facilities reported on 2,167 CABG procedures using rECG and 3,235 CABG procedures using spECG. At each time point considered, spECG was associated with significantly fewer SSIs (Table 2). The rECG 30-day and 90-day SSI rates were similar to those found in the 5% sample, and may indicate that these Medicare facilities that did switch were previously representative of Medicare facilities as a whole. For the subsequent cost-effectiveness analysis, we use the lowest rate of SSI events identified in the Medicare analysis: 5.49% by 90 days post event.

**Table 2:** Analysis of medicare facilities switching from rECG to spECG during 2011–2014.

**Parameter**	**rECG**	**spECG**	**%** **Reduction** **(*p*-value)**
CABG procedures, *N*	2,167	3,235	NA
SSI during index admission, *N* (%)	39 (1.8)	34 (1.1)	39 (0.02)
SSI at 30 days, *N* (%)	85 (3.9)	70 (2.2)	44 (<0.01)
SSI at 90 days, *N* (%)	122 (5.6)	126 (3.9)	30 (0.03)

The original data presented in Lankiewicz et al, (6), represented 27,296 CABG cases and 1,152 SSI events up to 90 days post procedure. We calculated the odds ratio (OR) to allow the data to be extrapolated for used in a clinical model. The mean (95% CI) OR was 0.74 (0.62–0.89), indicating a 26% reduction in the odds of an SSI with use of spECG.

### Cost-Effectiveness Analysis

With use of rECG, mean length of stay following CABG was 9.1 days of which 2.3 days were in the ICU. With transition to spECG, length of stay was 8.9 days, of which 2.3 days were in the ICU. In the first year, the total mean per patient cost of care was $35,539 with rECG and $35,038 with spECG, a reduction of $501. There was no difference in life expectancy between rECG and spECG, but QALYs were slightly increased with use of spECG (+0.008). The major driver of outcomes (both cost and QALYs) at 1-year was a reduction in inpatient and outpatient DSWIs. Results were similar when the full 40-year time horizon was applied, where spECG was found to save an average of $450 per patient across the total cost of care ($65,497 rECG vs. 65,048 spECG). Use of spECG was also associated with a minor increase in life expectancy (+0.003 years) and QALYs (+0.008).

**Figure 2:**
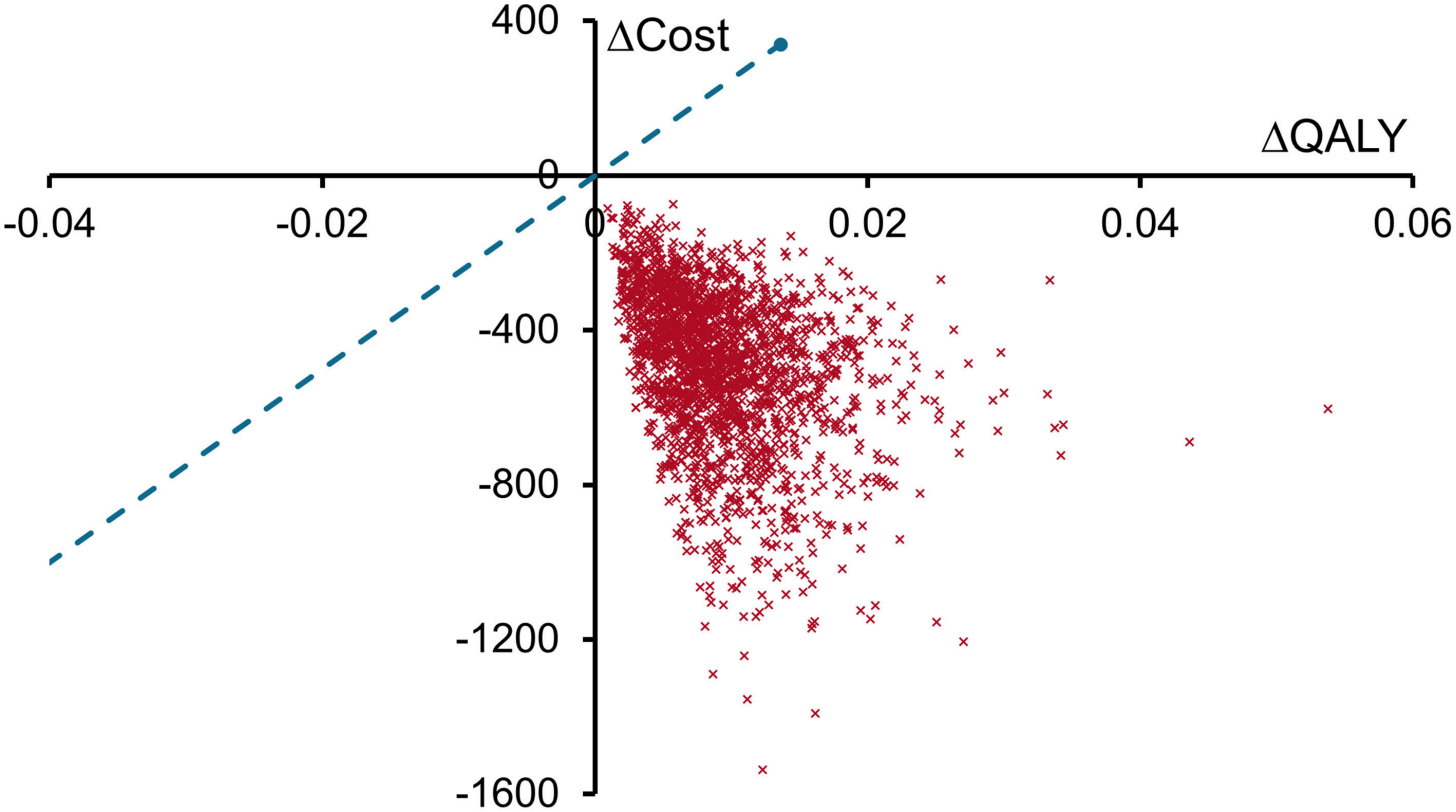
Cost-effectiveness plane for spECG vs. rECG. Each red cross represents the result of one of the 2,000 sensitivity analyses plotted as the change in QALYs with spECG (spECG—rECG) on the X-axis and the change in costs (2016 USD, $) with spECG on the Y-axis. The willingness to pay threshold (blue dashed line) is drawn at a value of $25,000 per QALY gained.

Reducing costs and increasing patient quality of life, spECG dominated rECG in this health economic analysis of cardiac monitoring associated with CABG procedures in the Medicare population. Sensitivity analyses found that both per patient cost savings and QALY increases with spECG were significant, having a mean (95% CrI) of $466 ($174 to $989) and 0.008 (0.002–0.021), respectively. The cost-effectiveness plane (Figure 2) demonstrated that all 2,000 simulations resulted in spECG dominating rECG. Given the circa 86,000 Medicare CABG procedures performed in 2014, Medicare may expect savings of $40 million ($15 million to $85 million) per year.

One-year life expectancy did not differ between rECG and spECG, but was lower with rECG than with spECG at year 40. This change was driven by the small number of SSIs and DSWIs that resulted in early patient mortality, the cumulative impact of which by year 40 was an extra 0.003 years of life with spECG. Although minor, this resulted in the per patient cost saving with spECG reducing from $501 at 1 year to $450 at 40 years. The so-called “survival paradox” meant that spECG patients incurred more standard care costs over years 2 to 40. These care costs depreciated the savings generated from the fewer inpatient and outpatient SSIs and DSWIs that spECG patients experienced in the first year. The analysis supports the conclusion that Medicare would expect to reduce costs if cardiac monitoring for CABG was switched from rECG to spECG. A small part of the early cost saving may, though, be reduced over the long term as patients live longer and require further treatment under the Medicare program. Sensitivity analyses found that these longer-term costs never fully negated the short term savings.

Sensitivity analyses considered the impact of uncertainty in input parameters on model outcomes, but individual hospitals can have substantial differences in costs and care practices. To account for a number of such possibilities, scenario analyses were performed. Reducing the SSI rate, eliminating DSWI, and halving the rate of prolonged mechanical ventilation all impacted outcomes but did not differ the conclusions drawn from the model (Table 3).

**Table 3:** Analyses testing potential differences in the base-case scenarios.

**Scenario**	**rECG** **cost**	**rECG** **QALY**	**spECG** **cost**	**spECG** **QALY**	**ICER for spECG vs. rECG**
Base case	65,497	7.232	65,048	7.240	Dominant
SSI rate: 2.5% after 90 days	64,543	7.249	64,339	7.253	Dominant
DSWI accounts for 0% of SSIs	64,580	7.244	64,367	7.249	Dominant
SSI: 2.5% and no DSWI	64,124	7.255	64,028	7.257	Dominant
2% of CABG patients require pMV	65,034	7.235	64,588	7.243	Dominant
Population: age 60 and 10% diabetes	75,982	10.167	75,567	10.175	Dominant
rECG at $4 per use	65,030	7.235	64,588	7.243	Dominant
spECG at $30 per use	65,034	7.235	64,618	7.243	Dominant

*CABG, Coronary artery bypass graft; Dominant, Lower cost and higher QALYs; DSWI, Deep sternal wound infection; ECG, Electrocardiogram; pMV, Prolonged mechanical ventilation; QALY, Quality-adjusted life years; rECG, Reusable ECG; spECG, Single-patient-use ECG; SSI, Surgical site infection; vs, versus*.

Although costs were not examined from the hospital perspective (charges), resource use can be used as a proxy for these costs. From the hospital perspective, minor reductions in ICU length of stay were identified. For every 100 patients using spECG as opposed to rECG, the hospital would be expected to avoid 2.1 ICU days and 12.8 hospital days. Furthermore, nursing burden may be reduced with spECG given that the model returned a mean of 93.4 fewer leads-off alarms per 100 patients assuming 8 days of ECG monitoring.

## Discussion

This analysis found that over the patient life times, the use of spECG resulted in a significant reduction in care costs and increased patient quality of life (QoL). The first year cost of care was $35,539 with rECG, the current standard of care. This is very close to the value published by Cohen et al. in their analysis of the SYNTAX trial, mean $33,190 ± 7,938 and median $30,903 (10). With respect to costs in the first year, our model used the initial cost of CABG provided by Cohen et al., but all other costs incurred in the first year (e.g., ICU days and readmissions) came from other publications. Others have published similar costs of care, with the ASCERT trial finding that CABG had an index hospitalization cost of $24,422 (30). These costs collected between 2004 and 2007, would be $32,265 in 2016 USD when using the same healthcare inflation index used in our analysis. Consistency of our rECG estimates with published clinical study data is encouraging for the application of our model.

The issue of cross-contamination due to reusable devices is not limited to cardiac surgery and is commonly associated with endoscopy. There is copious evidence of contamination of reusable endoscopes, duodenoscopes, and bronchocscopes (31–34). Real-world implications of this are demonstrated by the fact that removal of one of these devices was linked with the containment and end of an outbreak of *Pseudomonas aeruginosa* (32). Full and proper disinfection and reprocessing of reusable medical devices comes at cost (35), which is one item not considered in our analysis. Including these costs would, however, only increase the cost effectiveness of spECG, and so our results likely provide a conservative estimate of benefit.

Our analysis considers a Medicare population, with the rate of SSIs taken from analysis of the Medicare 5% professional sample from 2014. The impact of spECG on the rate of SSIs is also taken from this specific population. It is unknown whether the results presented here can be accurately extrapolated to other patient populations. Given that hospital facilities are commonly used jointly by both Medicare and private insurance, it seems logical that care practice would be equivalent and that results could be reasonably extended to other groups of patients. There are also other limitations to the study. Because this was a retrospective Medicare claims database analysis, facility-level data related to additional SSI reduction initiatives that may have occurred at the same time as the introduction of spECG cable and lead wire systems (and thus be a confounder in the analysis) were not available. The calculated SSI rate included only SSIs diagnosed or treated in an inpatient or outpatient hospital setting, and is thus likely to understate the full burden of SSIs in the community and to Medicare as the payer. A health economic model is simply a combination of mathematical formulae and no computational model can accurately recreate real life. The aim is to abstract real-world practice to a simplified but representative set of states and interventions that can be protocolized. In a cohort model such as the one presented here, individual patients characteristics are not available and as such risk factors are applied based on the “average” patient in the simulation. As individuals cannot be tracked and health states can be reentered, the number of events but not the number of patients having the event is available for analysis. As there is inherent uncertainty, individual model results are unlikely to be reproduced in a real-life setting. By testing the robustness of model outcomes to known uncertainties, a realistic range of potential results can be provided. It is therefore expected that use of spECG will result in cost savings of between $180 and $980 per patient for Medicare. Medicare savings will be derived from lower costs due to shorter hospital stays and fewer hospital readmissions.

Hospital savings were fewer ICU bed days and less time staff time required to attend leads-off alarms. Given the Medicare perspective taken, the impact on quality of care indicators on reimbursement was not modeled but it is unlikely that spECG would be detrimental in this regard. Readmission due to SSI is a quality of care indicator in the US, and the reduction in outpatient readmissions due to DSWI was found to be a substantial driver of outcomes in our analysis. Further work on specific outpatient SSI readmissions following CABG may be warranted given the results of our analysis and the use of this metric as a quality of care indicator.

## Conclusion

SSI following CABG remains a substantial burden within the Medicare population, impacting at least 5.49% of cases by 90 days post procedure. Transitioning to spECG from rECG within the Medicare population was found to be a cost-effective strategy, significantly reducing the cost of care and resulting in increased patient quality of life. In addition to benefits to patients and Medicare costs, it is also likely that hospitals could see a cost-savings benefit from introducing spECG for cardiac monitoring associated with CABG surgery.

## Data Availability

The datasets analyzed for this study is available from Medicare. The model developed is available from the authors on request.

## Author Contributions

JL conceived of the study, which was planned by RS. RS performed the literature searches, with articles reviewed by RS and JL. JL provided further analysis and raw data from a previous publication (6). The health economic model was designed and programmed by RS. Results were reviewed and interpreted by RS and JL. RS drafted the manuscript, which was reviewed and revised by JL. Both authors agreed to manuscript submission.

### Conflict of Interest Statement

RS is the owner of Coreva Scientific GmbH and Co KG, which received consultancy fees for performing, analyzing, and communicating the work presented here. JL is an employee of Cardinal Health.
