# Supplementary material for: The Cost Effectiveness of Single-Patient-Use Electrocardiograph Cable and Lead Systems in Monitoring for Coronary Artery Bypass Graft Surgery
Source: Front Cardiovasc Med. 2019 May 10;6:61. doi: 10.3389/fcvm.2019.00061 (PMC6523521; doi:10.3389/fcvm.2019.00061)
Supplement: Supplementary file 1 [file Data_Sheet_1.docx]

# Supplementary materials

The Cost Effectiveness Of Single-Patient-Use Electrocardiograph Cable And Lead Systems In Monitoring For Coronary Artery Bypass Graft Surgery

## Example structured search

The structured search used to identify comparative studies of electrocardiogram (ECG) leads and wires is shown in Table S1.

Table S1 Structured search in PubMed to identify relevant data on ECGleads

| Index | Aim | Search string | Hits |
| --- | --- | --- | --- |
| 1 | All comparative studies | "Randomized Controlled Trials as Topic"[Mesh] OR "Randomized Controlled Trial"[Publication Type] OR "Clinical Trial" [Publication Type] OR RCT[tiab] OR ((randomised[tiab] OR randomized[tiab] OR clinical[tiab] OR prospective[tiab] OR retrospective[tiab]) AND (controlled[tiab] OR controled[tiab] OR placebo[tw] OR matched[tiab]) AND (trial[tiab] OR trials[tiab] OR study[tiab] OR studies[tiab] OR cohort[tiab] OR comparison[tiab] OR control[tiab])) OR "Comparative Study"[Publication Type] OR "Meta-Analysis"[Publication Type] OR meta-analysis[tw] OR (indirect[tiab] AND “treatment comparison”[tiab]) OR "Prospective Studies"[Mesh] | 2,977,118 |
| 2 | All electrocardiogram studies | (Electrocardiogram[tw] OR Electrocardiography[tw] OR Electrocardiographic[tiab] OR ECG[tiab] OR "Disposable Equipment/adverse effects"[Mesh] OR "Disposable Equipment/instrumentation"[Mesh] OR "Disposable Equipment/statistics and numerical data"[Mesh] OR "Electrocardiography/instrumentation"[Mesh] OR "Electrocardiography/mortality"[Mesh] OR "Electrocardiography/surgery"[Mesh] OR "Electrocardiography/therapeutic use"[Mesh] OR "Equipment Reuse"[Mesh] OR "Telemetry/instrumentation"[Mesh] OR "Telemetry/mortality"[Mesh] OR "Telemetry/therapeutic use"[Mesh] OR "Telemetry/therapy"[Mesh]) AND (lead[tiab] OR leads[tiab] OR wire[tiab] OR wires[tiab] OR connector[tiab] OR connectors[tiab] OR reusable[tiab] OR disposable[tiab] OR “single use”[tiab] OR “single-use”[tiab]) | 24,048 |
| 3 | CABG, coronary surgery, or ICU studies | (("cardiac"[tiab] OR "heart"[tiab] OR "coronary"[tiab] OR "Open-Heart"[tiab]) AND (Surgery[tiab] OR bypass[tiab])) OR “coronary artery bypass grafting"[tw] OR CABG[tiab] OR "Coronary Artery Bypass"[Mesh] OR "Coronary Artery Bypass/adverse effects"[Mesh] OR “intensive care”[tiab] OR ICU[tiab] OR “acute care”[tiab] OR “critical care”[tiab] OR ((cardiac[tiab] OR cardiology[tiab]) AND (unit[tiab] OR units[tiab])) | 328,228 |
| 4 | All studies since 2007 | "2007/01/01"[PDAT]: "2018/01/01"[PDAT] | 10,134,122 |
| 5 | Recent comparative studies of ECG leads | #1 AND #2 AND #3 AND #4 | 153 |
| 6 | Those in English | #5 AND english[lang] | 145 |

## Table S2: Full parameter list

| Parameter | Base case | Variance | Reference |  |
| --- | --- | --- | --- | --- |
| Time horizon | 40 |  |  |  |
| Currency | USD |  |  |  |
| Year for currency | 2016 |  |  |  |
| QALY discount rate, % per year | 3.5 |  | Moore MA, Boardman AE, Vining AR. The choice of the social discount rate and the opportunity cost of public funds. J Benefit-Cost Anal 2013;4:401–9. |  |
| Cost discount rate, % per year | 3.5 |  | Moore MA, Boardman AE, Vining AR. The choice of the social discount rate and the opportunity cost of public funds. J Benefit-Cost Anal 2013;4:401–9. |  |
| Age, years | 73 | 2.29592 | McNeely C, Markwell S, Vassileva C. Trends in Patient Characteristics and Outcomes of Coronary Artery Bypass Grafting in the 2000 to 2012 Medicare Population. Ann Thorac Surg 2016;102:132–8. |  |
| Gender, % female | 29.8 | 0.1774 | McNeely C, Markwell S, Vassileva C. Trends in Patient Characteristics and Outcomes of Coronary Artery Bypass Grafting in the 2000 to 2012 Medicare Population. Ann Thorac Surg 2016;102:132–8. |  |
| Obese, % patients | 35 | 0.48424 | Ghanta RK, LaPar DJ, Zhang Q, et al. Obesity increases risk-adjusted morbidity, mortality, and cost following cardiac surgery. J Am Heart Assoc. 2017;6(3). |  |
| Morbid obesity, % patients | 6 | 0.24111 | Ghanta RK, LaPar DJ, Zhang Q, et al. Obesity increases risk-adjusted morbidity, mortality, and cost following cardiac surgery. J Am Heart Assoc. 2017;6(3). |  |
| Diabetes, % patients | 45.2 | 0.19304 | McNeely C, Markwell S, Vassileva C. Trends in Patient Characteristics and Outcomes of Coronary Artery Bypass Grafting in the 2000 to 2012 Medicare Population. Ann Thorac Surg 2016;102:132–8. |  |
| Purchase cost of lead (SOC), $ | 75 | 2 | https://www.medscape.com/viewarticle/819134, accessed 08 January 2018 |  |
| Number of patient uses (SOC) | 20 | 3.82653 | Assumption |  |
| Purchase cost of cable (SOC), $ | 160 | 10 | https://www.cablesandsensors.com/products/medtronic-physio-control-compatible-direct-connect-ekg-cable?variant=33805653768, accessed 08 January 2018 |  |
| Number of patient uses (SOC) | 30 | 5.10204 | Assumption |  |
| Cost per patient use (SOC) | 9.0833 | 1.65816 | Calculated value |  |
| Efficacy and safety | Reference |  |  |  |
| Kendall^TM^ DL purchase cost | 15 | 2 | Data on file with Cardinal health |  |
| Kendall ^TM^ DL surgical site infection RR | 0.74 | 0.08526 | Data on file with Cardinal health and currently in publication |  |
| Kendall ^TM^ DL false alarms RR | 0.81 | 0.13273 | Albert NM, Murray T, Bena JF, et al. Differences in Alarm Events Between Disposable and Reusable Electrocardiography Lead Wires. Am J Crit Care. 2015;24(1):67-74. |  |
| Kendall ^TM^ DL leads-off alarms, RR | 0.71 | 0.15154 | Albert NM, Murray T, Bena JF, et al. Differences in Alarm Events Between Disposable and Reusable Electrocardiography Lead Wires. Am J Crit Care. 2015;24(1):67-74. |  |
| Time on MV, days | 0.5 | 0.0001 | Raza S, Sabik JF, Ainkaran P, Blackstone EH. Coronary artery bypass grafting in diabetics: A growing health care cost crisis. J Thorac Cardiovasc Surg. 2015;150(2):304-312.e2. |  |
| ICU time, days | 1 | 0.0001 | Raza S, Sabik JF, Ainkaran P, Blackstone EH. Coronary artery bypass grafting in diabetics: A growing health care cost crisis. J Thorac Cardiovasc Surg. 2015;150(2):304-312.e2. |  |
| Hospital time, days | 8 | 1.78571 | McNeely C, Markwell S, Vassileva C. Trends in Patient Characteristics and Outcomes of Coronary Artery Bypass Grafting in the 2000 to 2012 Medicare Population. Ann Thorac Surg 2016;102:132–8. |  |
| Prolonged MV, % patients | 10.6 | 0.0001 | Ghanta RK, LaPar DJ, Zhang Q, et al. Obesity increases risk-adjusted morbidity, mortality, and cost following cardiac surgery. J Am Heart Assoc. 2017;6(3). |  |
| ECG monitoring, days | 8 | 1.78571 | McNeely C, Markwell S, Vassileva C. Trends in Patient Characteristics and Outcomes of Coronary Artery Bypass Grafting in the 2000 to 2012 Medicare Population. Ann Thorac Surg 2016;102:132–8.. |  |
| Home discharge, % patients | 74.5 | 0.16905 | McNeely C, Markwell S, Vassileva C. Trends in Patient Characteristics and Outcomes of Coronary Artery Bypass Grafting in the 2000 to 2012 Medicare Population. Ann Thorac Surg 2016;102:132–8. |  |
| Care facility discharge, % patients | 25.5 | 0.001 | McNeely C, Markwell S, Vassileva C. Trends in Patient Characteristics and Outcomes of Coronary Artery Bypass Grafting in the 2000 to 2012 Medicare Population. Ann Thorac Surg 2016;102:132–8. |  |
| Facility stay, days | 27 | 11.9133 | Grabowski DC. POST-ACUTE AND LONG-TERM CARE: A PRIMER ON SERVICES, EXPENDITURES AND PAYMENT METHODS 2010. |  |
| Reoperation rates, % patients | 5 | 1.97 | Lee MS, Kapoor N, Jamal F, Czer L, Aragon J, Forrester J, et al. Comparison of coronary artery bypass surgery with percutaneous coronary intervention with drug-eluting stents for unprotected left main coronary artery disease. J Am Coll Cardiol 2006;47:864–70. |  |
| In-hospital mortality, % patients by N days | 2.7 | 0.06287 | McNeely C, Markwell S, Vassileva C. Trends in Patient Characteristics and Outcomes of Coronary Artery Bypass Grafting in the 2000 to 2012 Medicare Population. Ann Thorac Surg 2016;102:132–8. |  |
| Mortality after N days | 13 | 0.0001 | McNeely C, Markwell S, Vassileva C. Trends in Patient Characteristics and Outcomes of Coronary Artery Bypass Grafting in the 2000 to 2012 Medicare Population. Ann Thorac Surg 2016;102:132–8. |  |
| Surgical site infections, % patients by N days | 5.49 | 0.5 | Data on file with Cardinal Health and currently in publication |  |
| SSI after N days | 90 | 0.0001 | Data on file with Cardinal Health and currently in publication |  |
| DSWI, % of SSIs | 40.75 | 2.64162 | Selby, L. V et al. Comparing surgical infections in National Surgical Quality Improvement Project and an Institutional Database. J. Surg. Res. 196, 416–20 (2015). (PMID: 25840487) |  |
| False alarms, per 100 monitoring days | 97.9 | 1.43384 | Albert NM, Murray T, Bena JF, et al. Differences in Alarm Events Between Disposable and Reusable Electrocardiography Lead Wires. Am J Crit Care. 2015;24(1):67-74. |  |
| Leads-off alarms, per 100 monitoring days | 40.9 | 4.91649 | Albert NM, Murray T, Bena JF, et al. Differences in Alarm Events Between Disposable and Reusable Electrocardiography Lead Wires. Am J Crit Care. 2015;24(1):67-74. |  |
| Background mortality, life expectancy | Life tables split by age and gender | | https://www.cdc.gov/nchs/data/nvsr/nvsr65/nvsr65_08.pdf accessed: January 08, 2018 |  |
| Length of stay in ICU, days given morbidly obese | 4 | 5.10204 | Ghanta RK, LaPar DJ, Zhang Q, et al. Obesity increases risk-adjusted morbidity, mortality, and cost following cardiac surgery. J Am Heart Assoc. 2017;6(3). |  |
| DSWI, RR given morbidly obese | 6.45 | 0.40438 | Ghanta RK, LaPar DJ, Zhang Q, et al. Obesity increases risk-adjusted morbidity, mortality, and cost following cardiac surgery. J Am Heart Assoc. 2017;6(3). |  |
| Prolonged MV, RR given morbidly obese | 1.73 | 0.10223 | Ghanta RK, LaPar DJ, Zhang Q, et al. Obesity increases risk-adjusted morbidity, mortality, and cost following cardiac surgery. J Am Heart Assoc. 2017;6(3). |  |
| Hospital mortality, RR given morbidly obese | 1.64 | 0.18231 | Ghanta RK, LaPar DJ, Zhang Q, et al. Obesity increases risk-adjusted morbidity, mortality, and cost following cardiac surgery. J Am Heart Assoc. 2017;6(3). |  |
| DSWI, RR given diabetes | 1.71 | 0.20408 | Raza S, Sabik JF, Ainkaran P, Blackstone EH. Coronary artery bypass grafting in diabetics: A growing health care cost crisis. J Thorac Cardiovasc Surg. 2015;150(2):304-312.e2. doi:10.1016/j.jtcvs.2015.03.041. |  |
| CABG cost, $ | 10,244 | 2663.69 | Cohen DJ, Osnabrugge RL, Magnuson EA, Wang K, Li H, Chinnakondepalli K, et al. Cost-effectiveness of percutaneous coronary intervention with drug-eluting stents versus bypass surgery for patients with 3-vessel or left main coronary artery disease final results from the synergy between percutaneous coronary intervention with TAXUS an. Circulation 2014;130:1146–57. |  |
| Mechanical ventilation cost, $ | 755.75 | 210.48-906.25 | Gershengorn HB, Garland A, Gong MN. Patterns of daily costs differ for medical and surgical intensive care unit patients. Ann Am Thorac Soc 2015;12:1831–6. |  |
| ICU per day, $ | 2535.52 | 2197.45-3066.62 | Gershengorn HB, Garland A, Gong MN. Patterns of daily costs differ for medical and surgical intensive care unit patients. Ann Am Thorac Soc 2015;12:1831–6.. |  |
| General ward per day, $ | 2357.3 | 235.73 | The Kaiser Family Foundation State Health Facts. Data Source: Hospital Adjusted Expenses per Inpatient Day. Data located at http://kff.org/health-costs/state-indicator/expenses-per-inpatient-day and last accessed January 08, 20187 |  |
| Nurse time, $ per hour | 57.93 | 4.57398 | Lord LM. Maintaining Hydration and Tube Patency in Enteral Tube Feedings. Safe Pract Patient Care 2006;5. |  |
| Care facility, $ per day | 420.43 | 413.334 | Grabowski DC. POST-ACUTE AND LONG-TERM CARE: A PRIMER ON SERVICES, EXPENDITURES AND PAYMENT METHODS 2010. |  |
| Patient at home, $ | 0 | 5 | Assumption |  |
| Days of home care, $ | 0 | 5 | Assumption |  |
| Inpatient SSI, $ per day | -157.6 | 209.551 | Shepard J, Ward W, Milstone A, Carlson T, Frederick J, Hadhazy E, et al. Financial Impact of Surgical Site Infections on Hospitals. JAMA Surg 2013;148:907. |  |
| Outpatient care for SSI, $ | 2582.6 | 838.406 | Olsen MA, Tian F, Wallace AE, Nickel KB, Warren DK, Fraser VJ, et al. Use of Quantile Regression to Determine the Impact on Total Health Care Costs of Surgical Site Infections Following Common Ambulatory Procedures. Ann Surg 2017;265:331–9. |  |
| Inpatient DSWI $ per day | -157.6 | 209.551 | Shepard J, Ward W, Milstone A, Carlson T, Frederick J, Hadhazy E, et al. Financial Impact of Surgical Site Infections on Hospitals. JAMA Surg 2013;148:907. |  |
| Readmission for DSWI, $ | 23,585 | 6815.23 | Nabagiez JP, Shariff MA, Molloy WJ, Demissie S, McGinn JT. Cost Analysis of Physician Assistant Home Visit Program to Reduce Readmissions After Cardiac Surgery. Ann Thorac Surg 2016;102:696–702. |  |
| False alarm, minutes to investigate | 2 | 0.2 |  |  |
| Leads-off alarm, minutes to investigate | 1 | 0.1 |  |  |
| SSI, additional days in hospital | 13.3 | 20.8 | Alasmari, F. A. et al. Temporal trends in the incidence of surgical site infections in patients undergoing coronary artery bypass graft surgery: A population-based cohort study, 1993 to 2008. Mayo Clin. Proc. 87, 1054–1061 (2012). (PMID: 23127732) |  |
| DSWI, additional days in hospital | 24 | 5 | Sears ED, Wu L, Waljee JF, Momoh AO, Zhong L, Chung KC. The Impact of Deep Sternal Wound Infection on Mortality and Resource Utilization: A Population-based Study. World J Surg. 2016;40(11):2673-2680. |  |
| Mortality during CABG, % of patients | 0.61 | 0.00057 | Mazzeffi M, Zivot J, Buchman T, Halkos M. In-hospital mortality after cardiac surgery: patient characteristics, timing, and association with postoperative length of intensive care unit and hospital stay. Ann Thorac Surg 2014;97:1220–5. |  |
| Prolonged MV, days | 7.9 | 8.5 | Widyastuti Y, Stenseth R, Pleym H, Wahba A, Videm V. Pre-operative and intraoperative determinants for prolonged ventilation following adult cardiac surgery. Acta Anaesthesiol Scand 2012;56:190–9. |  |
| Mortality cost, $ | 0 | 100 | Assumption |  |
| Baseline QALY | 0.85 | 0.158 | Cohen DJ, Osnabrugge RL, Magnuson EA, Wang K, Li H, Chinnakondepalli K, et al. Cost-effectiveness of percutaneous coronary intervention with drug-eluting stents versus bypass surgery for patients with 3-vessel or left main coronary artery disease final results from the synergy between percutaneous coronary intervention with TAXUS an. Circulation 2014;130:1146–57. |  |
| Annual QALY decrement | 0.0034 | 0.00306-0.00374 | Szende A, et al. Self-Reported Population Health: An International Perspective based on EQ-5D. Springer 2014. ISBN 978-94-007-7596-1 (eBook) |  |
| CABG, QALY | 0.741 | 0.191 | Cohen DJ, Osnabrugge RL, Magnuson EA, Wang K, Li H, Chinnakondepalli K, et al. Cost-effectiveness of percutaneous coronary intervention with drug-eluting stents versus bypass surgery for patients with 3-vessel or left main coronary artery disease final results from the synergy between percutaneous coronary intervention with TAXUS an. Circulation 2014;130:1146–57. |  |
| Mechanical ventilation (MV), QALY | -0.39 | -0.59-0.09 | Vainiola T, Roine RP, Pettilä V, et al. Effect of health-related quality-of-life instrument and quality-adjusted life year calculation method on the number of life years gained in the critical care setting. Value Heal 2011;14:1130–4. |  |
| ICU stay, QALY | 0.402 | 0.3618-0.4422 | Normilio-Silva, K. et al. Long-Term Survival, Quality of Life, and Quality-Adjusted Survival in Critically Ill Patients With Cancer*. Crit. Care Med. 44, 1327–1337 (2016). |  |
| General ward stay, QALY | 0.52 | 0.45-0.59 | Marti J, Hall P, Hamilton P, et al. One-year resource utilisation, costs and quality of life in patients with acute respiratory distress syndrome (ARDS): secondary analysis of a randomised controlled trial. J Intensive Care 2016;4:56. |  |
| Care facility, QALY | 0.53 | 0.03 | Lung T, Howard K, Etherton-Beer C, Sim M, Lewin G, Arendts G. Comparison of the HUI3 and the EQ-5D-3L in a nursing home setting. PLoS One 2017;12:1–10. |  |
| SSI, QALY | 0.198 | 0.04-0.8 | [1] Lee BY, Wiringa AE, Bailey RR, Goyal V, Lewis GJ, Tsui BYK, et al. Screening cardiac surgery patients for MRSA: an economic computer model. Am J Manag Care 2010;16:e163–73. and [2] Gheorghe A, Moran G, Duffy H, Roberts T, Pinkney T, Calvert M. Health Utility Values Associated with Surgical Site Infection: A Systematic Review. Value Heal 2015;18:1126–37. |  |
| DSWI, QALY | 0.198 | 0.04-0.8 | [1] Lee BY, Wiringa AE, Bailey RR, Goyal V, Lewis GJ, Tsui BYK, et al. Screening cardiac surgery patients for MRSA: an economic computer model. Am J Manag Care 2010;16:e163–73. and [2] Gheorghe A, Moran G, Duffy H, Roberts T, Pinkney T, Calvert M. Health Utility Values Associated with Surgical Site Infection: A Systematic Review. Value Heal 2015;18:1126–37. |  |
| Future care costs, $ per year | 4,673 | 524.14 | Cohen DJ, Osnabrugge RL, Magnuson EA, Wang K, Li H, Chinnakondepalli K, et al. Cost-effectiveness of percutaneous coronary intervention with drug-eluting stents versus bypass surgery for patients with 3-vessel or left main coronary artery disease final results from the synergy between percutaneous coronary intervention with TAXUS an. Circulation 2014;130:1146–57. |  |
| Decrement, future care costs | 92.29 | 215.39 | Cohen DJ, Osnabrugge RL, Magnuson EA, Wang K, Li H, Chinnakondepalli K, et al. Cost-effectiveness of percutaneous coronary intervention with drug-eluting stents versus bypass surgery for patients with 3-vessel or left main coronary artery disease final results from the synergy between percutaneous coronary intervention with TAXUS an. Circulation 2014;130:1146–57. |  |

BMI, Body mass index; CABG, Coronary artery bypass graft; DSWI, Deep sternal wound infection; ICU, Intensive care unit; MV, Mechanical ventilation; QALY, Quality-adjusted life expectancy; SSI, Surgical site infection
